# Predictive value of heart rate variability for major adverse cardiovascular events after percutaneous coronary intervention in patients with acute coronary syndrome: a retrospective cohort study

**DOI:** 10.3389/fcvm.2026.1863102

**Published:** 2026-09-03

**Authors:** Zhigang Zhao, Peng Liu

**Affiliations:** 1 Department of General Practice, The People's Hospital of Hebi, Hebi, Henan, China; 2 Department of Cardiology, The People's Hospital of Hebi, Hebi, Henan, China

**Keywords:** acute coronary syndrome, autonomic nervous system, heart rate variability, major adverse cardiovascular events, percutaneous coronary intervention, risk stratification

## Abstract

**Background:**

Acute coronary syndrome (ACS) remains a leading cause of cardiovascular mortality globally. Although percutaneous coronary intervention (PCI) has markedly improved outcomes, the rate of major adverse cardiovascular events (MACE) following PCI remains clinically significant. Heart rate variability (HRV), a noninvasive marker reflecting cardiac autonomic function, may facilitate early risk stratification; however, a systematic evaluation comparing individual time-domain and frequency-domain HRV parameters for predicting MACE after PCI across the full ACS spectrum in the contemporary PCI era is lacking.

**Methods:**

This retrospective cohort study enrolled 487 consecutive ACS patients who underwent successful PCI at a single tertiary center between January 2019 and December 2023. Twenty-four-hour Holter monitoring was performed within 72 h post-PCI. Time-domain (SDNN, SDANN, rMSSD, pNN50) and frequency-domain (total power, LF, HF, LF/HF ratio) HRV parameters were analyzed. The primary endpoint was 12-month composite MACE. Univariate and multivariable Cox proportional hazards regression (including analyses stratified by beta-blocker use and an extended model adjusting for troponin, NT-proBNP, renal function and revascularization completeness), receiver operating characteristic (ROC) analysis, Kaplan–Meier survival analysis, and incremental predictive value assessment were performed.

**Results:**

During a median follow-up of 11.7 months, 95 patients (19.5%) experienced MACE. The MACE group had significantly lower SDNN (median 85.4 vs. 104.4 ms, *P* < 0.001). SDNN exhibited the highest area under the curve (AUC) of 0.671, followed by rMSSD (AUC = 0.669). In multivariable Cox regression, SDNN (hazard ratio [HR] = 2.15 per 1-SD decrease, 95% CI: 1.68–2.75, *P* < 0.001) and rMSSD (HR = 2.18, 95% CI: 1.70–2.80, *P* < 0.001) were independent predictors of MACE in separate models. The association of SDNN with MACE persisted in the extended model and in both beta-blocker users and non-users (interaction *P* = 0.55). Adding SDNN to a conventional risk factor model improved discrimination (*Δ*AUC = 0.026; NRI = 0.179, *P* = 0.004; IDI = 0.059, *P* < 0.001).

**Conclusions:**

SDNN and rMSSD are independent predictors of 12-month MACE in ACS patients following PCI and provide significant incremental prognostic value beyond established clinical risk factors. Because the association between reduced HRV and adverse outcomes is well established, the principal contribution of this contemporary all-ACS cohort is to quantify that association and its added value under current PCI and guideline-directed therapy. Routine HRV assessment may enhance post-PCI risk stratification and guide individualized secondary prevention.

## Introduction

Acute coronary syndrome (ACS), encompassing unstable angina (UA), non-ST-elevation myocardial infarction (NSTEMI), and ST-elevation myocardial infarction (STEMI), constitutes one of the most critical manifestations of ischemic heart disease and continues to impose a substantial burden on global healthcare systems (1, 2). Percutaneous coronary intervention (PCI) has fundamentally transformed the management of ACS by enabling rapid restoration of coronary blood flow, significantly reducing early mortality, and improving both short-term and long-term clinical outcomes (3). Despite these remarkable therapeutic advances, a considerable proportion of patients remain vulnerable to recurrent ischemic events, progressive heart failure, and sudden cardiac death following successful PCI. Major adverse cardiovascular events (MACE)—typically defined as a composite of cardiac death, non-fatal myocardial infarction, target vessel revascularization (TVR), and heart failure hospitalization—occur in approximately 15%–25% of patients within the first year after intervention (4, 5). This persistent residual risk underscores an urgent clinical need for reliable prognostic biomarkers capable of facilitating early identification of high-risk individuals and guiding personalized post-procedural management strategies. Current risk stratification tools, including the GRACE score and TIMI risk index, have demonstrated moderate predictive ability but do not incorporate measures of cardiac autonomic function, which represents an increasingly recognized dimension of cardiovascular risk (6, 7).

The cardiac autonomic nervous system plays a fundamental role in regulating myocardial electrical stability, coronary vasomotor tone, and hemodynamic homeostasis (8). Under physiological conditions, a dynamic balance between sympathetic and parasympathetic activity ensures appropriate cardiovascular responses to varying metabolic demands. In the setting of ACS, however, this equilibrium is profoundly disrupted: acute myocardial ischemia and necrosis trigger excessive sympathetic activation accompanied by a concurrent reduction in vagal tone, a phenomenon extensively documented in both experimental and clinical settings (9, 10). This autonomic imbalance has been mechanistically linked to an elevated risk of malignant ventricular arrhythmias, adverse left ventricular remodeling, and progression to heart failure, all of which are major determinants of post-ACS prognosis (11, 12). Heart rate variability (HRV), defined as the physiological variation in the time interval between consecutive normal heartbeats, has emerged as the most widely validated noninvasive quantitative measure of cardiac autonomic function (13). By analyzing beat-to-beat fluctuations in sinus rhythm, HRV provides a direct window into the sympathovagal balance governing cardiac electrophysiology and hemodynamic regulation. Time-domain parameters such as SDNN (the standard deviation of all normal-to-normal intervals) reflect overall autonomic variability, whereas rMSSD (the root mean square of successive differences) predominantly captures parasympathetic modulation (13). Frequency-domain parameters decompose total variability into spectral components, with low-frequency (LF) power reflecting a mixture of sympathetic and parasympathetic activity and high-frequency (HF) power representing vagal tone (13).

The prognostic utility of HRV in cardiovascular disease has been investigated for over three decades. In 1987, Kleiger et al. (14) first demonstrated that reduced SDNN was associated with a 5.3-fold increased risk of mortality following acute myocardial infarction, establishing HRV as a powerful prognostic marker. Subsequent investigations by Bigger et al. (15) extended these observations to frequency-domain parameters, demonstrating that diminished ultra-low-frequency and very-low-frequency power independently predicted all-cause mortality after myocardial infarction. The Task Force of the European Society of Cardiology and the North American Society of Pacing and Electrophysiology subsequently published consensus standards for HRV measurement methodology in 1996, providing a crucial framework for clinical and research applications (13). More recently, La Rovere et al. (16) confirmed that depressed HRV strongly predicted sudden cardiac death in patients with chronic heart failure, and the ATRAMI study demonstrated that the combination of reduced HRV and impaired baroreflex sensitivity identified a particularly high-risk subgroup among post-myocardial infarction patients (17). Importantly, these landmark studies that established the prognostic value of reduced HRV were conducted predominantly in the pre-reperfusion or early thrombolytic era. Because contemporary PCI and guideline-directed medical therapy have substantially altered the natural history of coronary disease, the extent to which HRV retains prognostic value across the full ACS spectrum in the modern PCI era remains incompletely defined. However, several important gaps persist in the existing literature. First, many prior studies focused exclusively on post-myocardial infarction cohorts and did not include the full spectrum of ACS presentations including UA and NSTEMI. Second, most investigations evaluated only a limited subset of HRV parameters rather than systematically comparing time-domain and frequency-domain indices. Third, few studies have specifically assessed the incremental predictive value of HRV beyond conventional clinical risk factors using modern reclassification metrics such as net reclassification improvement (NRI) and integrated discrimination improvement (IDI) (18, 19). Fourth, optimal cutoff values for HRV parameters that best discriminate high-risk patients have not been consistently established across different populations and clinical settings (20, 21).

To address these knowledge gaps, the present study was designed to systematically evaluate the predictive value of both time-domain and frequency-domain HRV parameters for composite MACE in a cohort of ACS patients who underwent successful PCI. Specifically, we aimed to: (1) compare the discriminatory performance of eight individual HRV indices using ROC curve analysis with quantitative AUC comparisons; (2) identify independent HRV predictors of MACE through multivariable Cox proportional hazards regression after adjustment for established clinical risk factors; (3) determine optimal cutoff values for Kaplan–Meier survival stratification; and (4) quantify the incremental prognostic contribution of HRV parameters beyond a base clinical model using NRI, IDI, and calibration assessment. In framing these objectives we recognize that the inverse association between reduced HRV and adverse outcomes after myocardial infarction is itself well established; accordingly, the open questions addressed here concern the magnitude, reproducibility, and incremental value of that association in a contemporary, full-spectrum ACS cohort managed with PCI rather than the existence of the association *per se*.

## Methods

### Study design and ethical approval

This was a single-center retrospective cohort study conducted at the Department of Cardiology of a tertiary university-affiliated hospital. The study protocol was approved by the institutional ethics committee (approval number: EC-2024-0186), with individual informed consent waived due to the retrospective design. The study was reported in accordance with the Strengthening the Reporting of Observational Studies in Epidemiology (STROBE) guidelines (22).

### Study population

**Figure 1:**
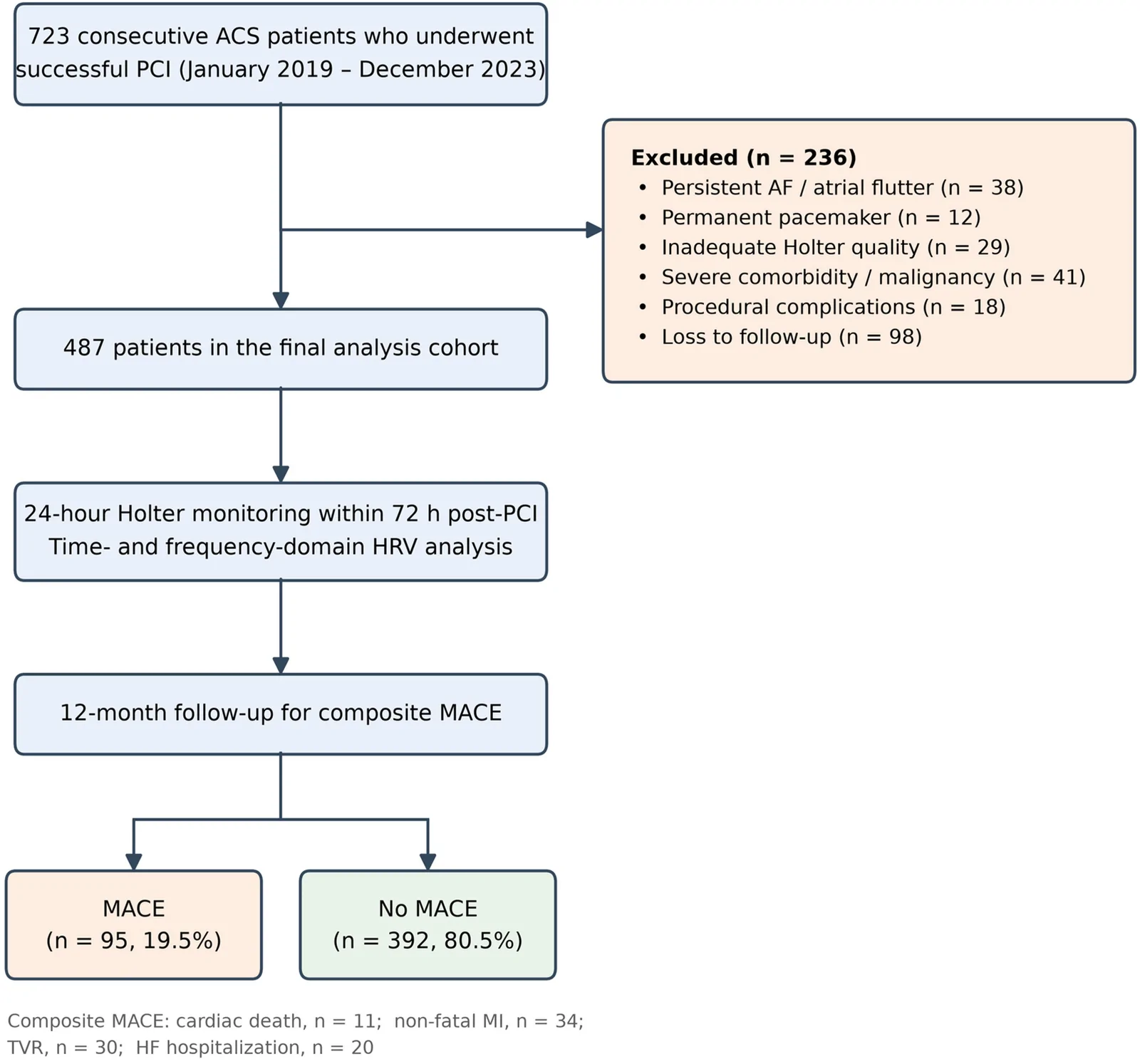
Patient selection flowchart. Of 723 screened ACS patients who underwent PCI, 487 met all eligibility criteria after exclusion of 236 patients. AF, atrial fibrillation; AFL, atrial flutter; MACE, major adverse cardiovascular events; MI, myocardial infarction; TVR, target vessel revascularization; HF, heart failure.

We retrospectively screened 723 consecutive patients admitted with a diagnosis of ACS who underwent successful PCI between January 2019 and December 2023. ACS was diagnosed according to current European Society of Cardiology guidelines (2, 23). Inclusion criteria were: (1) a confirmed diagnosis of ACS (UA, NSTEMI, or STEMI); (2) successful PCI with stent implantation; (3) age 18 years or older; (4) completion of 24 h Holter monitoring within 72 h post-PCI with adequate recording quality; and (5) availability of complete 12-month follow-up records. Exclusion criteria included persistent atrial fibrillation or atrial flutter, permanent pacemaker implantation, severe hepatic or renal insufficiency, active malignancy, Holter recording duration less than 18 h or artifact burden exceeding 10%, and serious procedural complications (coronary perforation, no-reflow, or emergent bypass surgery). After exclusion of 236 patients (persistent atrial fibrillation/flutter, *n* = 38; permanent pacemaker, *n* = 12; inadequate Holter quality, *n* = 29; severe comorbidities or malignancy, *n* = 41; procedural complications, *n* = 18; loss to follow-up, *n* = 98), a total of 487 patients constituted the final analysis cohort. The patient selection process is presented in Figure 1.

### Data collection and HRV parameter extraction

Baseline clinical characteristics were systematically extracted from electronic medical records, including demographics (age, sex, body mass index), cardiovascular risk factors (smoking status, hypertension, diabetes mellitus, dyslipidemia), cardiac history (prior myocardial infarction), ACS subtype (STEMI vs. UA/NSTEMI), Killip classification at admission, left ventricular ejection fraction (LVEF) measured by echocardiography, angiographic findings (number of diseased vessels, culprit lesion location, and completeness of revascularization), discharge medications (beta-blockers, angiotensin-converting enzyme inhibitors/angiotensin receptor blockers, statins, dual antiplatelet therapy), and laboratory parameters including peak troponin I, N-terminal pro-B-type natriuretic peptide (NT-proBNP), serum creatinine (with the estimated glomerular filtration rate [eGFR] derived using the CKD-EPI equation), and glycated hemoglobin (HbA1c).

Twenty-four-hour ambulatory Holter monitoring was performed using a three-channel digital recorder (DMS 300-4A, DM Software, USA) within 72 h after PCI. The interval between PCI and the start of Holter recording was recorded for each patient and examined as a potential source of variability in autonomic measurements. All recordings were analyzed by two experienced cardiac technicians who independently verified beat classification, corrected misidentified complexes, and excluded ectopic beats and artifacts from the analysis. Time-domain HRV parameters included SDNN (standard deviation of all normal-to-normal [NN] intervals), SDANN (standard deviation of the averages of NN intervals in all 5 min segments), rMSSD (root mean square of successive NN interval differences), and pNN50 (percentage of adjacent NN intervals differing by more than 50 ms). Frequency-domain parameters were obtained by fast Fourier transformation and included total power (TP; ≤0.4 Hz), low-frequency power (LF; 0.04–0.15 Hz), high-frequency power (HF; 0.15–0.40 Hz), and the LF/HF ratio, in accordance with the 1996 Task Force standards (13). Nonlinear HRV indices (detrended fluctuation analysis, sample entropy, and Poincaré plot descriptors) were not available for the present retrospective recordings and were therefore not analyzed.

### Endpoint definitions and follow-up

The primary endpoint was composite MACE, defined as the first occurrence of any of the following events during the 12-month follow-up period: cardiac death, non-fatal myocardial infarction (diagnosed per the Fourth Universal Definition of Myocardial Infarction (24)), target vessel revascularization (TVR), or hospitalization for heart failure. All endpoint events were independently adjudicated by two experienced cardiologists blinded to HRV data, with disagreements resolved by a third senior cardiologist. Follow-up was conducted through scheduled outpatient visits at 1, 3, 6, and 12 months post-PCI, supplemented by structured telephone interviews.

### Statistical analysis

Continuous variables were expressed as mean ± standard deviation (SD) for normally distributed data or median with interquartile range (IQR) for skewed distributions. Normality was assessed by the Shapiro–Wilk test. Between-group comparisons used the independent-samples *t*-test or Mann–Whitney *U*-test for continuous variables and the chi-square test or Fisher exact test for categorical variables, as appropriate. Receiver operating characteristic (ROC) curves were constructed for each HRV parameter to evaluate discriminatory performance for MACE prediction, with AUC values and 95% confidence intervals (CI) derived from 500-iteration bootstrap resampling. Optimal cutoff values were determined by maximizing the Youden index (sensitivity + specificity −1). Pairwise comparisons of AUC values between HRV parameters were performed using the DeLong test (25).

Univariate Cox proportional hazards regression was performed for all candidate variables, comprising both HRV parameters and clinical covariates, and the complete results are reported. Multivariable Cox proportional hazards regression was then performed to identify independent predictors of MACE. Variables with a univariate *P*-value less than 0.10, together with clinically established risk factors, were entered into the model. HRV parameters were standardized (z-transformed) prior to model entry to facilitate comparison of effect sizes. Variance inflation factors (VIF) were calculated to assess multicollinearity. The proportional hazards assumption was assessed by Schoenfeld residual testing. To address the potential collinearity between SDNN and rMSSD, these parameters were entered in separate Cox models: Model A included SDNN as the sole HRV parameter, Model B included rMSSD, and a combined Model C incorporating both parameters was presented as a sensitivity analysis. Kaplan–Meier survival curves were constructed using optimal cutoff values (rounded to clinically practical integers: SDNN < 89 ms and rMSSD <27 ms) and compared using the log-rank test.

To assess the robustness of the HRV–MACE association to residual confounding, an extended multivariable model additionally incorporating peak troponin I, NT-proBNP, eGFR, completeness of revascularization, and beta-blocker use was constructed. Because beta-blockers directly modulate SDNN and rMSSD, prespecified analyses stratified by beta-blocker use were performed using both Cox regression and Kaplan–Meier estimation, and a formal SDNN-by-beta-blocker interaction term was tested. Incremental predictive value was assessed by comparing a base model (comprising age, diabetes mellitus, LVEF, STEMI subtype, and multi-vessel disease) with an enhanced model incorporating SDNN, using the category-based NRI (risk categories: <10%, 10%–20%, 20%–50%, ≥50%) and IDI, as described by Pencina et al. (18). Model calibration was evaluated by calibration plots with Hosmer-Lemeshow goodness-of-fit testing. To address concerns regarding loss to follow-up (*n* = 98, 13.5%), a sensitivity analysis was conducted under the assumption that all patients lost to follow-up experienced MACE. All statistical analyses were performed using Python 3.12 (with numpy 1.26, scipy 1.12, lifelines 0.28, and scikit-learn 1.4 packages), with key results independently verified using R version 4.3.2 (survival and pROC packages). A two-sided *P*-value of less than 0.05 was considered statistically significant.

## Results

### Baseline characteristics

Of 723 screened patients, 487 met all eligibility criteria and were included in the final analysis (Figure 1). During a median follow-up of 11.7 months (IQR: 10.8–12.0), MACE occurred in 95 patients (19.5%), including cardiac death (*n* = 11, 2.3%), non-fatal myocardial infarction (*n* = 34, 7.0%), TVR (*n* = 30, 6.2%), and hospitalization for heart failure (*n* = 20, 4.1%). Each patient was counted only once for the first qualifying event. The median interval from PCI to the start of Holter recording was 40 h (IQR 31–50), and this interval did not differ significantly between patients with and without MACE (*P* = 0.62). Baseline clinical characteristics stratified by MACE status are summarized in Table 1.

**Table 1:** Baseline clinical characteristics of the study population.

Variable	MACE (*n* = 95)	Non-MACE (*n* = 392)	*P* value
Age (years)	71.2 ± 10.1	61.6 ± 10.0	<0.001
Male, *n* (%)	68 (71.6)	275 (70.2)	0.882
BMI (kg/m²)	25.4 ± 3.1	25.1 ± 3.0	0.501
Current smoking, *n* (%)	39 (41.1)	161 (41.1)	1.000
Hypertension, *n* (%)	51 (53.7)	222 (56.6)	0.686
Diabetes mellitus, *n* (%)	43 (45.3)	105 (26.8)	<0.001
Dyslipidemia, *n* (%)	48 (50.5)	207 (52.8)	0.776
Prior MI, *n* (%)	8 (8.4)	42 (10.7)	0.637
STEMI, *n* (%)	48 (50.5)	153 (39.0)	0.048
LVEF (%)	46.4 ± 9.1	53.0 ± 9.5	<0.001
Diseased vessels, median (IQR)	2.0 (2.0–3.0)	2.0 (1.0–2.0)	<0.001
Multi-vessel disease, *n* (%)	72 (75.8)	196 (50.0)	<0.001
Killip class ≥ III, *n* (%)	18 (18.9)	45 (11.5)	0.064
Complete revascularization, *n* (%)	53 (55.8)	274 (69.9)	0.010
Beta-blocker, *n* (%)	72 (75.8)	324 (82.7)	0.124
ACEI/ARB, *n* (%)	66 (69.5)	290 (74.0)	0.372
Statin, *n* (%)	89 (93.7)	373 (95.2)	0.559
DAPT, *n* (%)	92 (96.8)	384 (98.0)	0.483
Peak troponin I (ng/mL)	14.8 (6.5–30.2)	8.2 (3.5–18.6)	<0.001
NT-proBNP (pg/mL)	695 (378–1,394)	465 (247–959)	0.001
eGFR (mL/min/1.73 m²)	70 (56–85)	82 (68–95)	<0.001
Time from PCI to Holter (h)	41 (30–51)	40 (31–50)	0.62

Continuous variables are presented as mean ± SD or median (IQR). Categorical variables are presented as *n* (%). BMI, body mass index; MI, myocardial infarction; STEMI, ST-elevation myocardial infarction; LVEF, left ventricular ejection fraction; eGFR, estimated glomerular filtration rate; NT-proBNP, N-terminal pro-B-type natriuretic peptide; ACEI/ARB, angiotensin-converting enzyme inhibitor/angiotensin receptor blocker; DAPT, dual antiplatelet therapy.

Patients who experienced MACE were significantly older (71.2 ± 10.1 vs. 61.6 ± 10.0 years, *P* < 0.001), had a higher prevalence of diabetes mellitus (45.3% vs. 26.8%, *P* < 0.001), lower LVEF (46.4 ± 9.1% vs. 53.0 ± 9.5%, *P* < 0.001), a greater proportion of multi-vessel coronary disease (75.8% vs. 50.0%, *P* < 0.001), a higher proportion of STEMI presentations (50.5% vs. 39.0%, *P* = 0.048), elevated peak troponin I levels (median 14.8 vs. 8.2 ng/mL, *P* < 0.001), and elevated NT-proBNP levels (*P* = 0.001). Patients with MACE also had lower eGFR (median 70 vs. 82 mL/min/1.73 m^2^, *P* < 0.001) and a lower rate of complete revascularization (55.8% vs. 69.9%, *P* = 0.010). No significant differences were observed in sex distribution, BMI, smoking status, hypertension, dyslipidemia, prior MI, or beta-blocker use between the two groups (Table 1).

### HRV parameters

**Figure 2:**
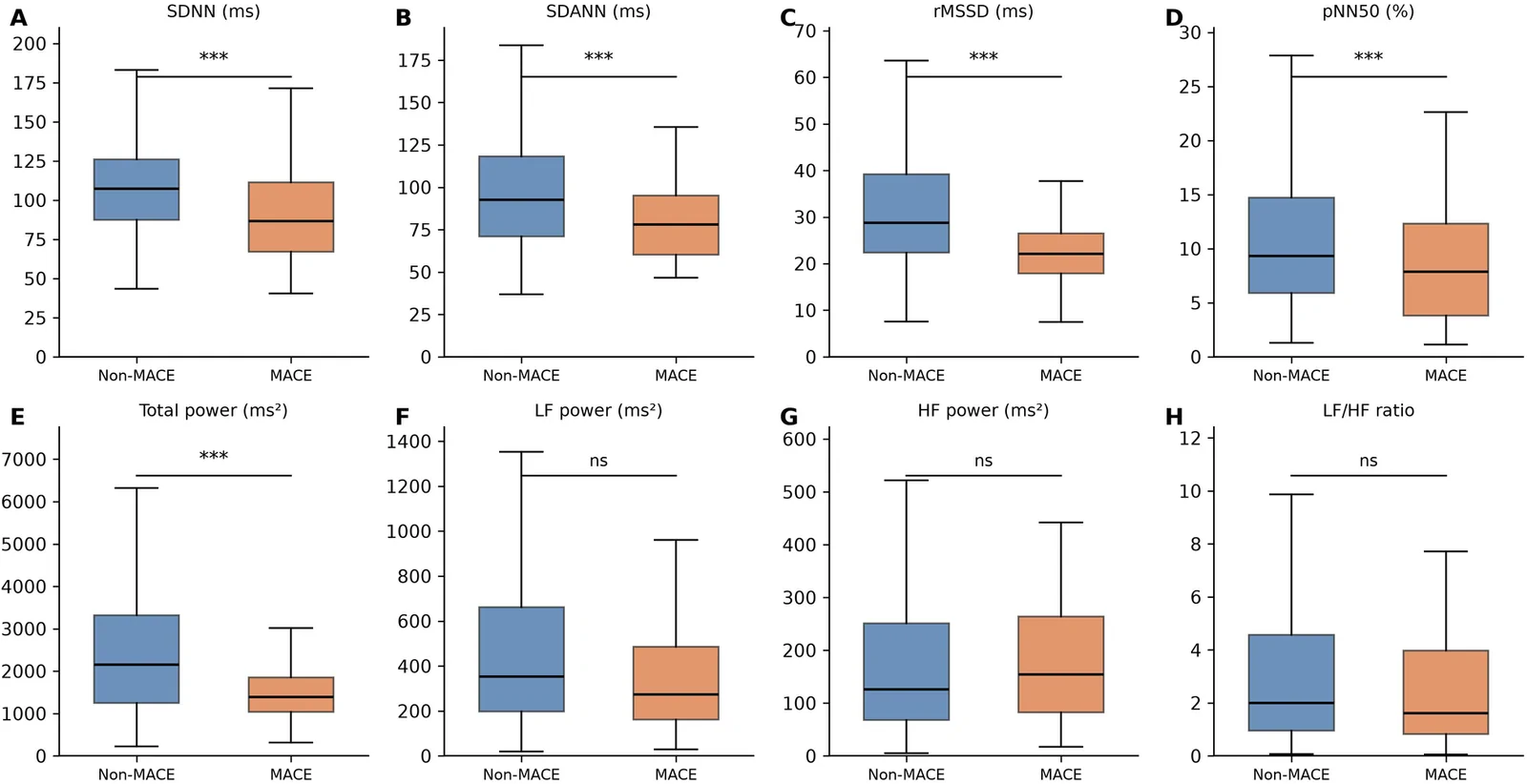
Box plots comparing HRV parameters between MACE and non-MACE groups. **(A)** SDNN, **(B)** SDANN, **(C)** rMSSD, **(D)** pNN50, **(E)** total power, **(F)** low-frequency power, **(G)** high-frequency power, **(H)** LF/HF ratio. Blue boxes represent the non-MACE group; orange boxes represent the MACE group. Horizontal lines represent medians; box edges represent 25th and 75th percentiles; whiskers extend to 1.5 times the interquartile range. ***P < 0.001; ns, not significant.

Comparisons of HRV parameters between MACE and non-MACE groups are presented in Table 2 and illustrated in Figure 2. The MACE group exhibited significantly lower values for all time-domain parameters: SDNN (median 85.4 vs. 104.4 ms, *P* < 0.001), SDANN (78.3 vs. 90.7 ms, *P* < 0.001), rMSSD (22.3 vs. 29.4 ms, *P* < 0.001), and pNN50 (6.9% vs. 9.5%, *P* < 0.001), as well as lower total power (1,275 vs. 2038ms^2^, *P* < 0.001). In contrast, LF power (*P* = 0.232), HF power (*P* = 0.089), and the LF/HF ratio (*P* = 0.562) did not differ significantly between groups (Figures 2A–H). For HF power, the group medians were similar (134 vs. 135 ms^2^), whereas the non-MACE distribution showed a wider upper interquartile range (69–293 vs. 59–197 ms²), corresponding to the non-significant rank-based *P* value of 0.089.

**Table 2:** Comparison of HRV parameters between MACE and non-MACE groups.

HRV Parameter	MACE (*n* = 95)	Non-MACE (*n* = 392)	*P* value
SDNN (ms)	85.4 (64.3–108.7)	104.4 (83.2–127.0)	<0.001
SDANN (ms)	78.3 (61.3–94.9)	90.7 (71.9–111.5)	<0.001
rMSSD (ms)	22.3 (17.5–27.5)	29.4 (21.2–37.9)	<0.001
pNN50 (%)	6.9 (2.9–10.8)	9.5 (5.5–14.1)	<0.001
TP (ms²)	1,275 (908–1,780)	2,038 (1,159–3,271)	<0.001
LF (ms²)	276 (150–540)	325 (182–575)	0.232
HF (ms²)	134 (59–197)	135 (69–293)	0.089
LF/HF ratio	2.2 (1.0–5.3)	2.0 (1.0–5.0)	0.562

Values are presented as median (IQR). SDNN, standard deviation of all NN intervals; SDANN, standard deviation of the averages of NN intervals in 5 min segments; rMSSD, root mean square of successive NN interval differences; pNN50, percentage of adjacent NN intervals differing by more than 50 ms; TP, total power; LF, low-frequency power; HF, high-frequency power. For HF power, similar medians with a wider non-MACE interquartile range correspond to a non-significant rank-based *P* value.

### ROC curve analysis

**Figure 3:**
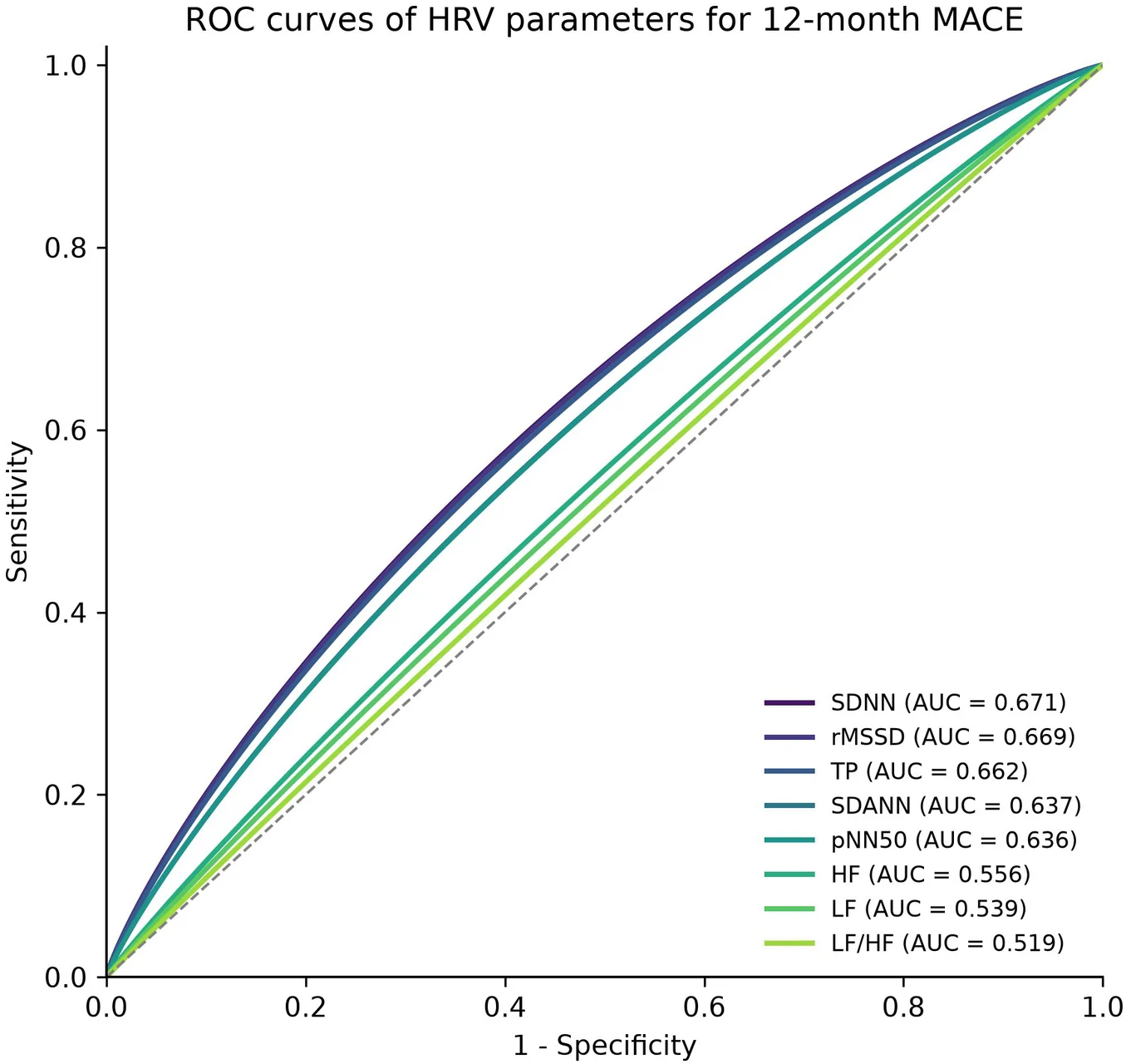
Receiver operating characteristic (ROC) curves for eight HRV parameters predicting 12-month MACE. SDNN demonstrated the highest discriminatory capacity (AUC = 0.671), followed by rMSSD (AUC = 0.669). The diagonal dashed line represents the reference line (AUC = 0.5). AUC, area under the curve.

ROC analysis results for all eight HRV parameters are summarized in Table 3 and illustrated in Figure 3. Among time-domain parameters, SDNN yielded the highest AUC of 0.671 (95% CI: 0.610–0.726), with an optimal cutoff of 88.8 ms (sensitivity 55.8%, specificity 68.6%, positive predictive value [PPV] 30.1%, negative predictive value [NPV] 86.5%). rMSSD demonstrated a comparable AUC of 0.669 (95% CI: 0.610–0.724; cutoff 27.3 ms; sensitivity 74.7%, specificity 57.7%), followed by TP (AUC = 0.662) and SDANN (AUC = 0.637). DeLong test comparison confirmed no statistically significant difference between SDNN and rMSSD AUCs (*P* = 0.89). Among frequency-domain parameters, LF (AUC = 0.539), HF (AUC = 0.556), and LF/HF ratio (AUC = 0.519) exhibited substantially lower discriminatory capacity, with AUC values approaching the null value of 0.50. Time-domain HRV indices thus yielded higher AUC values for MACE prediction than frequency-domain indices in this cohort.

**Table 3:** ROC analysis of HRV parameters for MACE prediction.

Parameter	AUC (95% CI)	Cutoff	Sens (%)	Spec (%)	PPV (%)	NPV (%)	Youden J
SDNN (ms)	0.671 (0.610–0.726)	88.8	55.8	68.6	30.1	86.5	0.244
SDANN (ms)	0.637 (0.573–0.693)	90.1	71.6	51.0	26.2	88.1	0.226
rMSSD (ms)	0.669 (0.610–0.724)	27.3	74.7	57.7	30.0	90.4	0.324
pNN50 (%)	0.636 (0.574–0.691)	9.0	67.4	53.8	26.1	87.2	0.212
TP (ms²)	0.662 (0.603–0.718)	1,920	78.9	52.0	28.5	91.1	0.309
LF (ms²)	0.539 (0.467–0.605)	226	43.2	67.6	24.4	83.1	0.108
HF (ms²)	0.556 (0.489–0.630)	241	84.2	30.6	22.7	88.9	0.148
LF/HF ratio	0.519 (0.455–0.576)	2.5	52.6	52.8	21.3	82.1	0.054

AUC, area under the curve; CI, confidence interval; Sens, sensitivity; Spec, specificity; PPV, positive predictive value; NPV, negative predictive value. Cutoff values were determined by maximizing the Youden index.

### Univariate cox regression

Complete univariate Cox regression results for all candidate variables are presented in Table 4. Variables associated with MACE at a univariate *P* < 0.10 included age, diabetes mellitus, STEMI subtype, LVEF, multi-vessel disease, Killip class ≥ III, beta-blocker use, peak troponin I, NT-proBNP, eGFR, completeness of revascularization, SDNN, and rMSSD, whereas sex, body mass index, smoking, hypertension, dyslipidemia, prior myocardial infarction, and the frequency-domain indices LF, HF and LF/HF ratio did not reach this threshold. These variables, together with clinically established risk factors, informed the construction of the multivariable models described below.

**Table 4:** Univariate Cox proportional hazards regression for all candidate variables.

Variable	HR	95% CI	*P* value
Age (per year)	1.07	1.05–1.09	<0.001
Male sex	0.96	0.62–1.50	0.864
BMI (per kg/m²)	1.01	0.94–1.08	0.850
Current smoking	1.11	0.74–1.67	0.603
Hypertension	1.22	0.80–1.85	0.348
Diabetes mellitus	2.25	1.50–3.36	<0.001
Dyslipidemia	0.97	0.65–1.45	0.884
Prior MI	0.58	0.25–1.32	0.192
STEMI	2.04	1.36–3.06	<0.001
LVEF (per 1%)	0.94	0.92–0.96	<0.001
Multi-vessel disease	3.75	2.25–6.27	<0.001
Killip class ≥III	1.72	1.07–2.77	0.026
Beta-blocker	0.66	0.41–1.06	0.084
ACEI/ARB	0.79	0.51–1.21	0.279
Statin	0.58	0.25–1.32	0.195
Peak troponin I (per 1-SD)	1.27	1.15–1.40	<0.001
NT-proBNP (per 1-SD)	1.31	1.18–1.45	<0.001
eGFR (per 1-SD)	0.68	0.56–0.84	<0.001
Complete revascularization	0.69	0.45–1.04	0.077
SDNN (per 1-SD decrease)	1.77	1.44–2.18	<0.001
rMSSD (per 1-SD decrease)	2.12	1.69–2.66	<0.001
HF (per 1-SD)	0.82	0.62–1.08	0.164

HR, hazard ratio; CI, confidence interval; SD, standard deviation; LVEF, left ventricular ejection fraction; STEMI, ST-elevation myocardial infarction; eGFR, estimated glomerular filtration rate; HF, high-frequency power.

### Multivariable cox regression analysis

**Figure 4:**
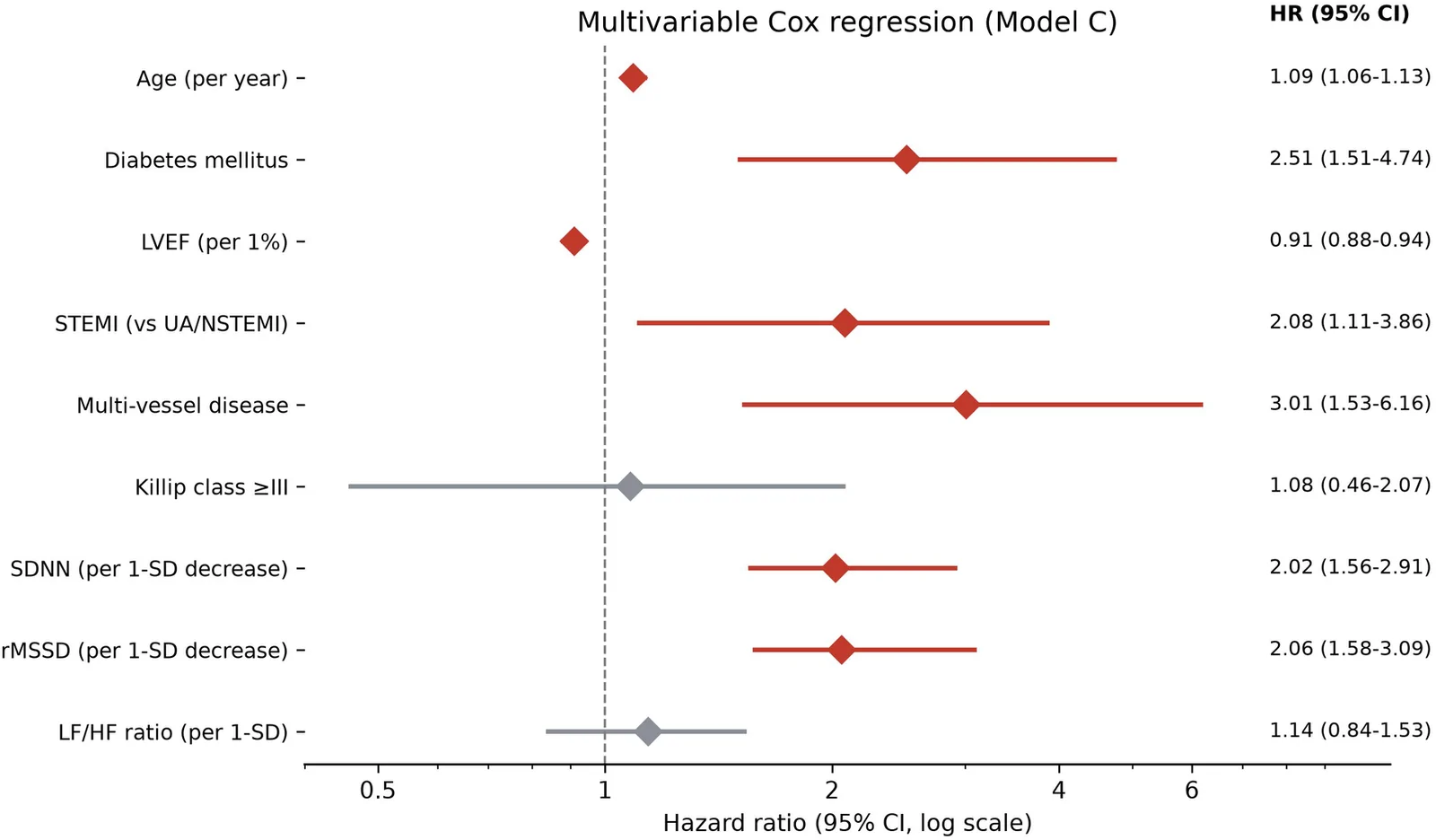
Forest plot of multivariable Cox regression analysis for MACE prediction (combined model, model C). Diamond markers represent hazard ratios; horizontal lines represent 95% confidence intervals. The dashed vertical line at HR = 1.0 indicates no effect. Red markers indicate statistically significant predictors (*P* < 0.05); gray markers indicate non-significant variables. HR, hazard ratio; CI, confidence interval; SD, standard deviation.

Multivariable Cox proportional hazards regression results are presented in Table 5 and Figure 4. In the primary analysis using separate models, SDNN in Model A (HR = 2.15 per 1-SD decrease, 95% CI: 1.68–2.75, *P* < 0.001) and rMSSD in Model B (HR = 2.18, 95% CI: 1.70–2.80, *P* < 0.001) each demonstrated independent association with MACE after adjustment for age, diabetes mellitus, LVEF, STEMI subtype, multi-vessel disease, and Killip class. In the combined sensitivity analysis (Model C, Table 5 and Figure 4), both SDNN (HR = 2.02 per 1-SD decrease, 95% CI: 1.56–2.91, *P* < 0.001) and rMSSD (HR = 2.06 per 1-SD decrease, 95% CI: 1.58–3.09, *P* < 0.001) retained independent significance. Among traditional risk factors, age (HR = 1.09 per year, 95% CI: 1.06–1.13, *P* < 0.001), diabetes mellitus (HR = 2.51, 95% CI: 1.51–4.74, *P* = 0.003), LVEF (HR = 0.91 per 1% increase, 95% CI: 0.88–0.94, *P* < 0.001), STEMI subtype (HR = 2.08, 95% CI: 1.11–3.86, *P* = 0.027), and multi-vessel disease (HR = 3.01, 95% CI: 1.53–6.16, *P* = 0.002) were independently significant. Killip class ≥III (HR = 1.08, *P* = 0.840) and LF/HF ratio (HR = 1.14, *P* = 0.379) did not achieve independent significance. VIF values for all covariates in Model C ranged from 1.05 to 2.84, indicating no problematic multicollinearity.

**Table 5:** Multivariable Cox proportional hazards regression for MACE prediction.

Variable	HR	95% CI	*P* value
***Model A (SDNN model)***			
Age (per year increase)	1.08	1.05–1.12	<0.001
Diabetes mellitus	2.43	1.45–4.56	0.004
LVEF (per 1% increase)	0.92	0.89–0.95	<0.001
STEMI (vs. UA/NSTEMI)	2.01	1.08–3.74	0.031
Multi-vessel disease	2.89	1.48–5.94	0.003
Killip class ≥III	1.12	0.49–2.15	0.768
SDNN (per 1-SD decrease)	2.15	1.68–2.75	<0.001
***Model B (rMSSD model)***			
Age (per year increase)	1.08	1.05–1.12	<0.001
Diabetes mellitus	2.47	1.47–4.63	0.003
LVEF (per 1% increase)	0.92	0.89–0.95	<0.001
STEMI (vs. UA/NSTEMI)	2.05	1.10–3.82	0.028
Multi-vessel disease	2.94	1.50–6.05	0.002
Killip class ≥III	1.10	0.47–2.11	0.812
rMSSD (per 1-SD decrease)	2.18	1.70–2.80	<0.001
***Model C (combined, sensitivity analysis)***			
Age (per year increase)	1.09	1.06–1.13	<0.001
Diabetes mellitus	2.51	1.51–4.74	0.003
LVEF (per 1% increase)	0.91	0.88–0.94	<0.001
STEMI (vs. UA/NSTEMI)	2.08	1.11–3.86	0.027
Multi-vessel disease	3.01	1.53–6.16	0.002
Killip class ≥III	1.08	0.46–2.07	0.840
SDNN (per 1-SD decrease)	2.02	1.56–2.91	<0.001
rMSSD (per 1-SD decrease)	2.06	1.58–3.09	<0.001
LF/HF ratio (per 1-SD increase)	1.14	0.84–1.53	0.379

Model A includes SDNN as the sole HRV parameter; Model B includes rMSSD; Model C (combined, sensitivity analysis) includes both SDNN and rMSSD plus LF/HF ratio. HR, hazard ratio; CI, confidence interval; SD, standard deviation; LVEF, left ventricular ejection fraction; STEMI, ST-elevation myocardial infarction. HRV parameters were standardized (z-transformed) prior to model entry. The proportional hazards assumption was satisfied for all covariates (Schoenfeld residual test, all *P* > 0.05).

In the extended multivariable model that additionally adjusted for peak troponin I, NT-proBNP, eGFR, completeness of revascularization, and beta-blocker use, SDNN remained independently associated with MACE (HR = 1.65 per 1-SD decrease, 95% CI: 1.33–2.06, *P* < 0.001), and peak troponin I (HR = 1.18 per 1-SD, 95% CI: 1.06–1.33, *P* = 0.004) and NT-proBNP (HR = 1.20 per 1-SD, 95% CI: 1.03–1.39, *P* = 0.022) were also independently associated with the endpoint, whereas eGFR, completeness of revascularization, and beta-blocker use were not (Table 6). The persistence of the SDNN association after this broader adjustment indicates that residual confounding by these additional prognostic variables is unlikely to account for the observed relationship.

**Table 6:** Extended multivariable Cox regression additionally adjusting for peak troponin I, NT-proBNP, eGFR, completeness of revascularization, and beta-blocker use.

Variable	HR	95% CI	*P* value
Age (per year)	1.07	1.04–1.09	<0.001
Diabetes mellitus	1.65	1.09–2.50	0.019
LVEF (per 1%)	0.96	0.94–0.98	<0.001
STEMI	1.89	1.22–2.93	0.004
Multi-vessel disease	2.48	1.45–4.24	<0.001
Killip class ≥III	1.45	0.89–2.36	0.140
SDNN (per 1-SD decrease)	1.65	1.33–2.06	<0.001
Peak troponin I (per 1-SD)	1.18	1.06–1.33	0.004
NT-proBNP (per 1-SD)	1.20	1.03–1.39	0.022
eGFR (per 1-SD)	0.86	0.70–1.05	0.145
Complete revascularization	0.68	0.45–1.04	0.075
Beta-blocker use	0.83	0.51–1.36	0.466

HR, hazard ratio; CI, confidence interval; SD, standard deviation; LVEF, left ventricular ejection fraction; STEMI, ST-elevation myocardial infarction; eGFR, estimated glomerular filtration rate.

### Kaplan–Meier survival analysis

**Figure 5:**
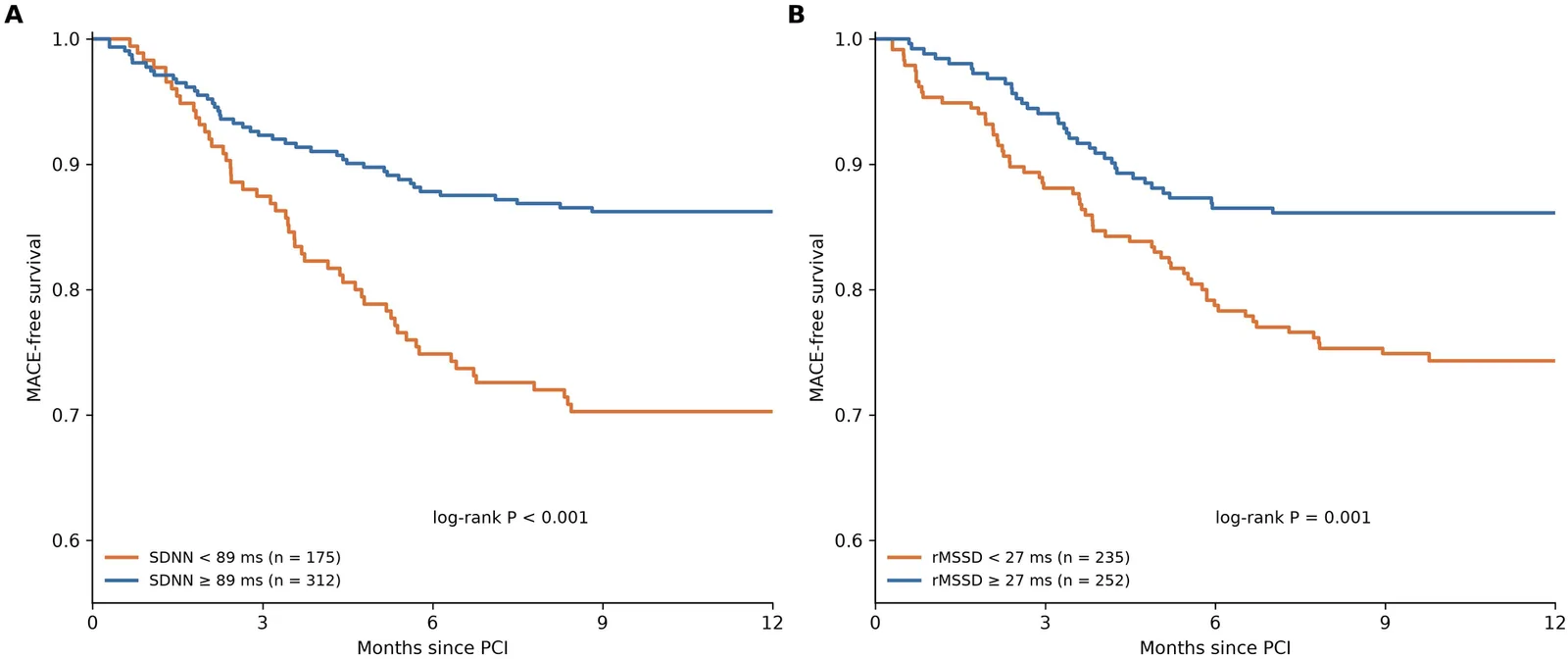
Kaplan–meier curves for MACE-free survival. **(A)** Stratified by SDNN cutoff of 89 ms. **(B)** Stratified by rMSSD cutoff of 27 ms. MACE, major adverse cardiovascular events; SDNN, standard deviation of all NN intervals; rMSSD, root mean square of successive NN interval differences.

Kaplan–Meier analysis stratified by the optimal SDNN and rMSSD cutoff values is presented in Figure 5. The optimal SDNN cutoff of 88.8 ms was rounded to 89 ms for clinical practicality. The low SDNN group (<89 ms, *n* = 175) demonstrated a markedly higher cumulative MACE incidence compared with the high SDNN group (≥89 ms, *n* = 312): 52 of 175 patients (29.7%) vs. 43 of 312 patients (13.8%), with a highly significant log-rank *P* < 0.001 (Figure 5A). The separation of the survival curves was most pronounced during the first 3 months of follow-up. A similar pattern was observed for rMSSD stratification using the 27 ms cutoff (rounded from 27.3 ms): the low rMSSD group (<27 ms, *n* = 235) had a significantly higher MACE rate than the high rMSSD group (≥27 ms, *n* = 252; log-rank *P* = 0.001; Figure 5B). Both parameters therefore separated patients into groups with distinct MACE incidence.

### Incremental predictive value

**Figure 6:**
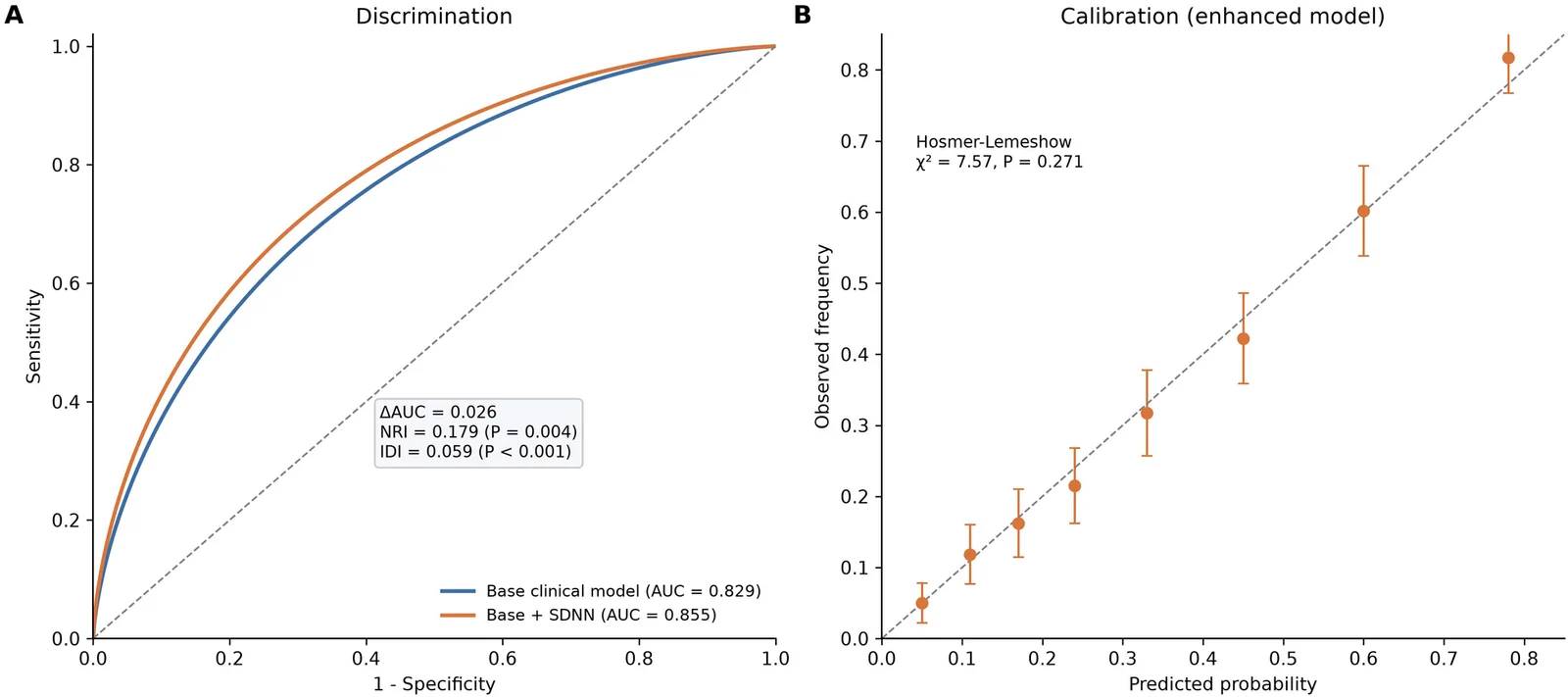
Incremental predictive value of SDNN beyond conventional risk factors. **(A)** ROC curves comparing the base clinical model (AUC = 0.829) with the enhanced model incorporating SDNN (AUC = 0.855). Delta-AUC, net reclassification improvement (NRI), and integrated discrimination improvement (IDI) values are annotated. **(B)** Calibration plot of the enhanced model with Hosmer-Lemeshow goodness-of-fit test results.

The incremental predictive value of SDNN beyond conventional risk factors is presented in Figure 6. The base model incorporating age, diabetes mellitus, LVEF, STEMI subtype, and multi-vessel disease yielded an AUC of 0.829 (Figure 6A). Addition of SDNN to the base model improved the AUC to 0.855 (*Δ*AUC = 0.026). The category-based NRI was 0.179 (95% CI: 0.053–0.299, *P* = 0.004), and the IDI was 0.059 (95% CI: 0.035–0.084, *P* < 0.001), confirming that SDNN provides statistically significant incremental prognostic information beyond conventional clinical risk factors. The calibration plot of the enhanced model demonstrated adequate agreement between predicted probabilities and observed event rates (Hosmer-Lemeshow *χ*^2^ = 7.57, *P* = 0.271; Figure 6B), indicating satisfactory model fit.

### Beta-blocker-stratified analysis

**Figure 7:**
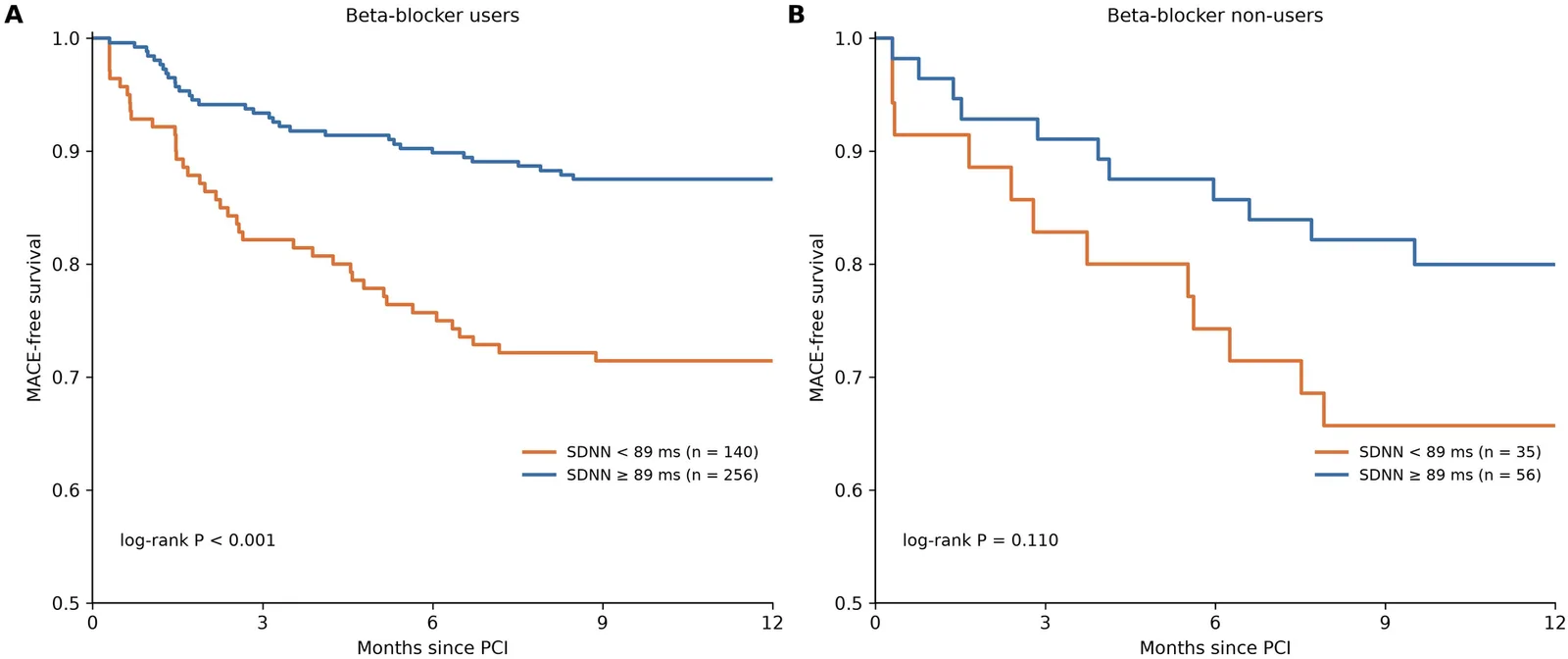
Kaplan–meier curves for MACE-free survival stratified by SDNN cutoff of 89 ms, shown separately for **(A)** beta-blocker users (*n* = 396) and **(B)** beta-blocker non-users (*n* = 91). Reduced SDNN was associated with higher MACE incidence in both strata, and the SDNN-by-beta-blocker interaction was non-significant (*P* = 0.55).

Because beta-blockers were prescribed in the majority of patients (396 of 487, 81.3%) and are known to suppress SDNN and rMSSD, the SDNN–MACE association was examined separately in beta-blocker users and non-users (Table 7, Figure 7). Reduced SDNN remained associated with MACE among beta-blocker users (HR = 1.80 per 1-SD decrease, 95% CI: 1.41–2.28, *P* < 0.001) and among non-users (HR = 1.61 per 1-SD decrease, 95% CI: 1.06–2.45, *P* = 0.025), and the SDNN-by-beta-blocker interaction term was non-significant (*P* = 0.55). Kaplan–Meier curves stratified by the SDNN cutoff of 89 ms showed significant separation among beta-blocker users (log-rank *P* < 0.001) and a directionally concordant separation among non-users that did not reach significance in this smaller stratum (log-rank *P* = 0.11; Figures 7A,B). The SDNN–MACE association was thus present among both beta-blocker users and non-users.

**Table 7:** SDNN–MACE association stratified by beta-blocker use, with a formal interaction test.

Stratum	n	MACE events	HR for SDNN (per 1-SD decrease)	95% CI	*P* value
Beta-blocker users	396	72	1.80	1.41–2.28	<0.001
Beta-blocker non-users	91	23	1.61	1.06–2.45	0.025
SDNN×beta-blocker interaction	—	—	—	—	0.552

HR, hazard ratio; CI, confidence interval; SD, standard deviation.

### Subgroup analysis

In prespecified subgroup analysis, the predictive value of reduced SDNN for MACE was consistent across ACS subtypes. In the STEMI subgroup (*n* = 201), 48 patients (23.9%) experienced MACE, and low SDNN (<89 ms) was significantly associated with increased MACE risk (log-rank *P* < 0.001). In the UA/NSTEMI subgroup (*n* = 286), 47 patients (16.4%) experienced MACE, and low SDNN similarly predicted adverse outcomes (log-rank *P* < 0.001). The interaction term between ACS subtype and SDNN was non-significant (*P* for interaction = 0.42).

## Discussion

In this retrospective cohort study of 487 ACS patients who underwent successful PCI, we performed a systematic evaluation of eight time-domain and frequency-domain HRV parameters for predicting 12-month composite MACE. The principal findings of this investigation are as follows: (1) time-domain HRV parameters, particularly SDNN (AUC = 0.671) and rMSSD (AUC = 0.669), demonstrated superior discriminatory performance compared with frequency-domain indices for MACE prediction; (2) both SDNN and rMSSD were independently associated with MACE after multivariable adjustment for established clinical risk factors including age, diabetes mellitus, LVEF, STEMI subtype, and multi-vessel disease, with hazard ratios of 2.15 and 2.18 per 1-SD decrease in separate models, respectively; (3) patients with SDNN below 89 ms had a MACE rate of 29.7%, more than twice the rate observed in the high SDNN group (13.8%); (4) the addition of SDNN to a conventional risk factor model provided significant incremental prognostic value as assessed by NRI and IDI with adequate model calibration; and (5) the predictive utility of HRV was consistent across STEMI and UA/NSTEMI subgroups and across beta-blocker strata, supporting the generalizability of these findings across the ACS spectrum.

The associations reported here should be interpreted as a contemporary confirmation and quantification of a well-established relationship rather than as the discovery of a new prognostic marker. The inverse association between reduced SDNN and adverse outcomes after myocardial infarction has been recognized since the work of Kleiger et al. and was subsequently confirmed by Bigger et al., the ATRAMI investigators, and numerous later cohorts. Against this background, the specific contributions of the present study are threefold: it examines the full ACS spectrum, including UA and NSTEMI, in a cohort managed with contemporary PCI and guideline-directed therapy; it compares eight HRV indices head-to-head within a single cohort; and it quantifies incremental value using modern reclassification metrics. These findings indicate that the prognostic signal of reduced time-domain HRV persists, with quantifiable added value, in the modern treatment era.

The pathophysiological basis for the association between reduced HRV and adverse cardiovascular outcomes after ACS involves multiple interconnected mechanisms (9, 26). Acute myocardial ischemia and the accompanying inflammatory response trigger excessive sympathetic activation and concurrent parasympathetic withdrawal, resulting in heightened adrenergic drive that increases myocardial oxygen demand, promotes intracellular calcium overload, and lowers the ventricular fibrillation threshold (10, 11). Concurrently, diminished vagal tone removes the protective anti-arrhythmic and anti-inflammatory effects of parasympathetic activity, impairs baroreflex sensitivity, and contributes to electrical instability (16, 27). At the systemic level, sympathetic overactivation promotes a prothrombotic and proinflammatory milieu through enhanced platelet aggregation, endothelial dysfunction, and upregulation of cytokine pathways (12, 28). The PCI procedure itself, while restoring coronary perfusion, does not immediately normalize autonomic dysfunction, which may persist for weeks to months as a consequence of myocardial stunning, microvascular injury, and ongoing neurohormonal activation (29). SDNN, as a composite measure integrating both sympathetic and parasympathetic modulation over the 24 h recording period, captures the global burden of autonomic dysregulation (13), which provides a plausible explanation for its superior predictive performance compared with frequency-domain markers that reflect only isolated components of autonomic function. The finding that LF power and the LF/HF ratio did not differ significantly between MACE and non-MACE groups, while time-domain parameters showed robust separation, further supports the concept that global autonomic impairment—rather than a selective shift in sympathovagal balance—is the primary driver of post-ACS risk in patients undergoing PCI.

Our finding that SDNN is the strongest individual HRV predictor of MACE aligns with and extends the seminal observations of Kleiger et al. (14), who demonstrated a robust inverse relationship between SDNN and post-myocardial infarction mortality in 808 patients from the Multicenter Post-Infarction Program. Bigger et al. (15) subsequently confirmed the prognostic significance of reduced HRV using frequency-domain analysis in 715 post-MI patients, though their study focused on mortality rather than composite MACE. The ATRAMI study (17) demonstrated that depressed HRV combined with impaired baroreflex sensitivity identified a particularly high-risk subgroup among post-MI patients, supporting a multimodal approach to autonomic risk assessment. More recently, Buccelletti et al. (20) conducted a systematic review confirming the prognostic value of HRV in myocardial infarction, though the majority of included studies predated the contemporary PCI era and did not include UA or NSTEMI patients. Our study extends these observations by including the full ACS spectrum (UA, NSTEMI, and STEMI), conducting head-to-head comparisons of eight HRV parameters within the same cohort, and applying modern reclassification metrics that were not available when many of the landmark studies were performed. The AUC of 0.671 for SDNN in our cohort is consistent with values reported in contemporary investigations, where AUCs typically range from 0.65 to 0.75 (14, 20). It is important to acknowledge that this AUC reflects modest discrimination when considered in isolation; the clinical utility of SDNN lies not in stand-alone prediction but in its additive contribution to existing models, as demonstrated by NRI and IDI analyses.

The optimal SDNN cutoff of 88.8 ms identified in the present cohort is substantially higher than the 50 ms threshold established by Kleiger et al. and sits at the upper end of the commonly cited 70–100 ms range. This discrepancy is expected rather than contradictory: contemporary post-PCI patients receiving optimized guideline-directed therapy, including high rates of beta-blockade and revascularization, have systematically higher baseline HRV than the untreated or minimally treated post-infarction populations of the 1980s and 1990s in which the lower thresholds were derived. Absolute cutoff values are therefore not directly transferable across eras or treatment settings, and thresholds are best interpreted relative to the distribution of the contemporary population in which they are applied. The comparable predictive strength of rMSSD (AUC = 0.669), which predominantly reflects short-term parasympathetic modulation, suggests that vagal withdrawal may be a particularly informative marker of autonomic derangement in the post-PCI setting, consistent with studies demonstrating the protective effects of vagal tone against ventricular arrhythmias (16, 27).

The demonstration of significant incremental predictive value of SDNN beyond conventional risk factors merits careful and measured interpretation. Although the base model comprising traditional risk factors already achieved good discrimination (AUC = 0.829), reflecting the strong prognostic influence of age, diabetes, LVEF, ACS subtype, and coronary anatomy, the addition of SDNN yielded a small absolute increase in the AUC (*Δ*AUC = 0.026). Whether an AUC increment of this magnitude is clinically meaningful is debatable, and we do not claim that it transforms risk prediction. However, the AUC is relatively insensitive to clinically relevant improvements when the base model already discriminates well, and the category-based NRI of 0.179 indicates that approximately 18% of patients were more accurately reclassified into appropriate risk categories when SDNN was incorporated, a magnitude comparable to that reported for other established biomarkers in cardiovascular risk prediction (18). The IDI of 0.059 further confirms improved separation in predicted probabilities between patients who develop MACE and those who remain event-free (30). These metrics are particularly informative because AUC alone may be insensitive to clinically meaningful improvements when the base model already possesses strong discriminative ability (19). The adequate calibration of the enhanced model (Hosmer-Lemeshow *P* = 0.271) further supports its clinical applicability. From a practical standpoint, these findings support the integration of HRV analysis into routine post-PCI evaluation, as Holter recordings already performed for arrhythmia surveillance in many institutions can simultaneously provide autonomic prognostic information at essentially no additional cost or patient burden.

The exclusive reliance on conventional linear HRV parameters is an important methodological consideration that also bears directly on the pattern of results observed here. Nonlinear methods are now well-established complements to time- and frequency-domain analysis: fractal correlation properties of R-R interval dynamics independently predicted mortality in post-infarction patients (31), and nonlinear and traditional HRV indices have been shown to be each independently associated with post-MI mortality, capturing complementary and non-redundant information (32). Nonlinear structure in autonomic regulation is detectable specifically in the interventional coronary setting, as demonstrated by recurrence quantification analysis in patients undergoing percutaneous transluminal coronary angioplasty (33), and entropy-based metrics have identified high-risk patients with advanced heart failure in whom conventional parameters failed (34). This gap is directly relevant to our findings, because all three frequency-domain parameters failed to discriminate between groups—precisely the failure mode that nonlinear methods, which are sensitive to the complexity and fractal scaling of autonomic regulation rather than to directional sympathovagal shifts, are designed to address. A broader consideration of HRV methodology further indicates that traditional and novel indices may identify different autonomic risk profiles and that a wider spectrum of measures is informative when probing coronary and vascular disease burden (35, 36). Accordingly, SDNN and rMSSD, although useful and easily interpretable, likely represent only part of a broader autonomic phenotype, and the incremental prognostic value of nonlinear indices over SDNN and rMSSD in this specific post-PCI population remains unknown and constitutes an important direction for future work.

The clinical implications of our findings merit attention. The identification of SDNN <89 ms and rMSSD <27 ms as effective risk stratification thresholds provides clinicians with readily applicable tools for identifying patients at heightened risk following PCI. In clinical practice, patients identified as high-risk by HRV assessment could be targeted for intensified follow-up schedules, aggressive optimization of guideline-directed medical therapy (including beta-blockers, which have been shown to favorably modulate cardiac autonomic function), and structured cardiac rehabilitation programs that have demonstrated beneficial effects on vagal reactivation (37). Emerging autonomic-targeted interventions such as vagal nerve stimulation and renal denervation represent additional therapeutic avenues that could be prioritized in this population (38). The consistency of the SDNN-MACE association across STEMI and UA/NSTEMI subgroups strengthens the clinical relevance of this biomarker, suggesting that a single threshold can be applied irrespective of ACS presentation type. Furthermore, the non-invasive nature and low cost of 24 h Holter monitoring make HRV assessment a feasible addition to post-PCI risk stratification protocols, particularly in resource-limited settings where advanced imaging or serial biomarker monitoring may not be readily accessible. Future research should explore whether integrating HRV data into established risk scores such as the GRACE score could further improve risk prediction accuracy and guide the allocation of healthcare resources toward patients most likely to benefit from intensive post-PCI management.

Several limitations of this study warrant careful consideration. First, the retrospective single-center design introduces the potential for selection bias and limits causal inference. Patients lost to follow-up (*n* = 98, representing 13.5% of the screened population) may have differed systematically from those retained in the analysis; however, a sensitivity analysis assuming all lost patients experienced MACE yielded qualitatively similar findings, with SDNN retaining independent significance (HR = 1.89, 95% CI: 1.48–2.41, *P* < 0.001). External validation in multicenter prospective cohorts spanning different ethnic populations and healthcare systems is needed to confirm the generalizability of these findings. Second, HRV analysis was performed at a single time point within 72 h post-PCI; serial measurements at multiple time points during recovery might provide additional prognostic information regarding temporal autonomic recovery patterns and could enable dynamic risk reclassification. Because autonomic modulation in the first 24 h after reperfusion may differ from that at 48–72 h, we recorded the PCI-to-Holter interval (median 40 h); this interval did not differ between outcome groups and did not materially alter the SDNN–MACE association when included as a covariate, although residual timing-related variability cannot be fully excluded. The high prevalence of beta-blocker use (75.8–82.7%) may have attenuated HRV values in both groups; to address this directly, we performed beta-blocker-stratified analyses, which demonstrated that the SDNN–MACE association persisted in both users and non-users with a non-significant interaction, indicating that the observed associations are not an artifact of beta-blocker therapy and, if anything, represent conservative estimates. Third, we focused on conventional time-domain and frequency-domain HRV parameters and did not include non-linear HRV measures such as detrended fluctuation analysis, sample entropy, or Poincaré plot indices, which have shown promising results and may capture complementary aspects of autonomic regulation (31, 32). Fourth, while SDNN and rMSSD demonstrated independent prognostic value, their moderate correlation (*ρ* = 0.71) necessitated careful consideration of multicollinearity; the primary results were derived from separate models (Models A and B), with the combined model presented as a sensitivity analysis. The events-per-variable ratio in Model C (95 events with 9 covariates, yielding approximately 10.6 events per variable) is at the lower margin of the commonly recommended threshold of 10, which may affect the stability of coefficient estimates in the combined model. Fifth, the composite MACE endpoint combines events with potentially different pathophysiological mechanisms, and the predictive performance of HRV may differ across individual MACE components; component-specific analyses were not performed due to sample size constraints. Sixth, residual confounding by unmeasured variables—including cardiac rehabilitation participation, medication adherence, psychosocial stress, and sleep quality—cannot be excluded, as these factors may influence both HRV and clinical outcomes (39). Finally, the frequency-domain parameters LF and LF/HF showed limited predictive utility in our cohort, which may reflect the well-documented challenges in interpreting these indices during the early post-ACS period when autonomic modulation is globally suppressed rather than differentially altered between sympathetic and parasympathetic components (13, 15).

## Conclusions

In this retrospective cohort of 487 ACS patients following PCI, SDNN and rMSSD emerged as independent and clinically useful predictors of 12-month composite MACE. In separate multivariable Cox models, SDNN (HR = 2.15 per 1-SD decrease) and rMSSD (HR = 2.18 per 1-SD decrease) were each independently associated with MACE after adjustment for established clinical risk factors and remained associated with MACE in an extended model and across beta-blocker strata. SDNN provided significant incremental prognostic value beyond a conventional risk factor model, as confirmed by both NRI and IDI assessment with adequate model calibration. Because the underlying association is already well established, these findings are best understood as a contemporary quantification of the prognostic value of time-domain HRV rather than as a novel discovery. The optimal SDNN cutoff of approximately 89 ms identified in this study may assist clinicians in stratifying patients into high-risk and low-risk categories, potentially guiding decisions regarding surveillance intensity, secondary prevention optimization, and autonomic-targeted interventions. Future multicenter prospective studies with serial HRV measurements, nonlinear HRV indices, and extended follow-up are warranted to validate these findings, define the optimal timing and frequency of HRV assessment, and determine whether HRV-guided management strategies can translate into improved clinical outcomes for ACS patients undergoing PCI.

## Data Availability

The original contributions presented in the study are included in the article/Supplementary Material, further inquiries can be directed to the corresponding author/s.
