# Supplementary material for: Predictive value of heart rate variability for major adverse cardiovascular events after percutaneous coronary intervention in patients with acute coronary syndrome: a retrospective cohort study
Source: Front Cardiovasc Med. 2026 Sep 3;13:1863102. doi: 10.3389/fcvm.2026.1863102 (PMC13581866; doi:10.3389/fcvm.2026.1863102)
Supplement: Supplementary file 1 [file Table1.docx]

**STROBE Statement—Checklist for Cohort Studies**

*Manuscript ID 1863102. Item locations refer to the Clean Copy manuscript.*

| **Section / Topic** | **Item** | **Recommendation** | **Reported in** |
| --- | --- | --- | --- |
| Title and abstract | 1 | Informative title and structured abstract | Title; Abstract |
| **Introduction** |  |  |  |
| Background/rationale | 2 | Scientific background and rationale | Introduction, paras 1–3 |
| Objectives | 3 | Specific objectives / hypotheses | Introduction, final paragraph |
| **Methods** |  |  |  |
| Study design | 4 | Key elements of study design | Methods, Study Design |
| Setting | 5 | Setting, locations, relevant dates | Methods, Study Design & Population |
| Participants | 6 | Eligibility criteria; sources and selection | Methods, Study Population; Figure 1 |
| Variables | 7 | Outcomes, exposures, predictors, confounders | Methods, Data Collection; Endpoint Definitions |
| Data sources/measurement | 8 | Sources and methods of assessment (HRV, Holter) | Methods, Data Collection and HRV Parameter Extraction |
| Bias | 9 | Efforts to address potential sources of bias | Methods, Statistical Analysis; Limitations |
| Study size | 10 | How study size was arrived at | Methods, Study Population; Figure 1 |
| Quantitative variables | 11 | Handling of quantitative variables; cutoffs | Methods, Statistical Analysis |
| Statistical methods | 12 | All statistical methods, subgroups, sensitivity analyses | Methods, Statistical Analysis |
| **Results** |  |  |  |
| Participants | 13 | Numbers at each stage; flow diagram | Results, Baseline; Figure 1 |
| Descriptive data | 14 | Characteristics, confounders, follow-up | Results, Baseline; Table 1 |
| Outcome data | 15 | Numbers of outcome events | Results, Baseline (95 MACE) |
| Main results | 16 | Unadjusted and adjusted estimates with CIs | Results; Tables 4–7; Figures 3–7 |
| Other analyses | 17 | Subgroups, interactions, sensitivity analyses | Results, Beta-blocker/Subgroup; Table 7; Figure 7 |
| **Discussion** |  |  |  |
| Key results | 18 | Summary of key results with reference to objectives | Discussion, para 1 |
| Limitations | 19 | Limitations, sources of bias, direction | Discussion, Limitations paragraph |
| Interpretation | 20 | Cautious overall interpretation | Discussion |
| Generalisability | 21 | External validity | Discussion; Limitations |
| **Other information** |  |  |  |
| Funding | 22 | Source of funding and role of funders | Declarations, Funding |
