# Supplementary material for: Predictive value of heart rate variability for major adverse cardiovascular events after percutaneous coronary intervention in patients with acute coronary syndrome: a retrospective cohort study
Source: Front Cardiovasc Med. 2026 Sep 3;13:1863102. doi: 10.3389/fcvm.2026.1863102 (PMC13581866; doi:10.3389/fcvm.2026.1863102)
Supplement: Supplementary file 2 [file Table2.docx]

**Reference Verification Report**

**Manuscript ID 1863102 — verification of all 39 references**

| **#** | **Reference (corrected where applicable)** | **Status** | **Verification note** |
| --- | --- | --- | --- |
| 1 | GBD 2019 Diseases and Injuries Collaborators. Global burden of 369 diseases and injuries in 204 countries and territories, 1990–2019. Lancet. 2020;396(10258):1204–1222. | **Verified** | Indexed landmark GBD study (Lancet). |
| 2 | Collet JP, Thiele H, Barbato E, et al. 2020 ESC Guidelines for the management of ACS without persistent ST-segment elevation. Eur Heart J. 2021;42(14):1289–1367. | **Verified** | ESC guideline, correctly cited. |
| 3 | Neumann FJ, Sousa-Uva M, Ahlsson A, et al. 2018 ESC/EACTS Guidelines on myocardial revascularization. Eur Heart J. 2019;40(2):87–165. | **Verified** | ESC/EACTS guideline, correctly cited. |
| 4 | Jernberg T, Hasvold P, Henriksson M, Hjelm H, Thuresson M, Janzon M. Cardiovascular risk in post-myocardial infarction patients: nationwide real world data demonstrate the importance of a long-term perspective. Eur Heart J. 2015;36(19):1163–1170. | **CORRECTED** | Original entry (Kikkert et al., Catheter Cardiovasc Interv 2017) not verifiable — replaced with verified source (PMID 25586123). |
| 5 | Palmerini T, Biondi-Zoccai G, Della Riva D, et al. Stent thrombosis with drug-eluting and bare-metal stents: a network meta-analysis. Lancet. 2012;379(9824):1393–1402. | **Verified** | Re-checked this round — authentic (Lancet 2012). |
| 6 | Fox KA, Dabbous OH, Goldberg RJ, et al. Prediction of risk of death and MI in the six months after presentation with ACS (GRACE). BMJ. 2006;333(7578):1091. | **Verified** | GRACE registry, correctly cited. |
| 7 | Morrow DA, Antman EM, Charlesworth A, et al. TIMI risk score for STEMI. Circulation. 2000;102(17):2031–2037. | **Verified** | Correctly cited. |
| 8 | Shen MJ, Zipes DP. Role of the autonomic nervous system in modulating cardiac arrhythmias. Circ Res. 2014;114(6):1004–1021. | **Verified** | Correctly cited. |
| 9 | Schwartz PJ, La Rovere MT, Vanoli E. Autonomic nervous system and sudden cardiac death. Circulation. 1992;85(1 Suppl):I77–I91. | **Verified** | Correctly cited. |
| 10 | Goldberger JJ, Cain ME, Hohnloser SH, et al. AHA/ACCF/HRS scientific statement on noninvasive risk stratification. Circulation. 2008;118(14):1497–1518. | **Verified** | Correctly cited. |
| 11 | Vaseghi M, Shivkumar K. The role of the autonomic nervous system in sudden cardiac death. Prog Cardiovasc Dis. 2008;50(6):404–419. | **Verified** | Correctly cited. |
| 12 | Carney RM, Blumenthal JA, Stein PK, et al. Depression, heart rate variability, and acute myocardial infarction. Circulation. 2001;104(17):2024–2028. | **Verified** | Correctly cited. |
| 13 | Task Force of the ESC and NASPE. Heart rate variability: standards of measurement, physiological interpretation, and clinical use. Circulation. 1996;93(5):1043–1065. | **Verified** | Foundational HRV standard, correctly cited. |
| 14 | Kleiger RE, Miller JP, Bigger JT Jr, Moss AJ. Decreased heart rate variability and its association with increased mortality after acute MI. Am J Cardiol. 1987;59(4):256–262. | **Verified** | Landmark HRV paper, correctly cited. |
| 15 | Bigger JT Jr, Fleiss JL, Steinman RC, Rolnitzky LM, Kleiger RE, Rottman JN. Frequency domain measures of heart period variability and mortality after MI. Circulation. 1992;85(1):164–171. | **Verified** | Correctly cited. |
| 16 | La Rovere MT, Pinna GD, Maestri R, et al. Short-term HRV strongly predicts sudden cardiac death in chronic heart failure. Circulation. 2003;107(4):565–570. | **Verified** | Correctly cited. |
| 17 | La Rovere MT, Bigger JT Jr, Marcus FI, Mortara A, Schwartz PJ. Baroreflex sensitivity and heart-rate variability in prediction of total cardiac mortality after MI (ATRAMI). Lancet. 1998;351(9101):478–484. | **Verified** | ATRAMI, correctly cited. |
| 18 | Pencina MJ, D’Agostino RB Sr, D’Agostino RB Jr, Vasan RS. Evaluating the added predictive ability of a new marker: from AUC to reclassification and beyond. Stat Med. 2008;27(2):157–172. | **Verified** | Correctly cited. |
| 19 | Cook NR. Use and misuse of the ROC curve in risk prediction. Circulation. 2007;115(7):928–935. | **Verified** | Correctly cited. |
| 20 | Buccelletti E, Gilardi E, Scaini E, et al. Heart rate variability and myocardial infarction: systematic literature review and metanalysis. Eur Rev Med Pharmacol Sci. 2009;13(4):299–307. | **Verified** | Re-checked this round — authentic (PMID 19694345). |
| 21 | Lanza GA, Cianflone D, Rebuzzi AG, et al. Prognostic value of ventricular arrhythmias and heart rate variability in patients with unstable angina. Heart. 2006;92(8):1055–1063. | **Verified** | Re-checked this round — authentic. |
| 22 | von Elm E, Altman DG, Egger M, et al. The STROBE statement. Lancet. 2007;370(9596):1453–1457. | **Verified** | Reporting-guideline reference, correctly cited. |
| 23 | Ibanez B, James S, Agewall S, et al. 2017 ESC Guidelines for the management of AMI in patients presenting with STEMI. Eur Heart J. 2018;39(2):119–177. | **Verified** | ESC guideline, correctly cited. |
| 24 | Thygesen K, Alpert JS, Jaffe AS, et al. Fourth universal definition of myocardial infarction (2018). Circulation. 2018;138(20):e618–e651. | **Verified** | Correctly cited. |
| 25 | DeLong ER, DeLong DM, Clarke-Pearson DL. Comparing the areas under two or more correlated ROC curves: a nonparametric approach. Biometrics. 1988;44(3):837–845. | **Verified** | Correctly cited. |
| 26 | Thayer JF, Yamamoto SS, Brosschot JF. The relationship of autonomic imbalance, HRV and cardiovascular disease risk factors. Int J Cardiol. 2010;141(2):122–131. | **Verified** | Correctly cited. |
| 27 | Zipes DP, Rubart M. Neural modulation of cardiac arrhythmias and sudden cardiac death. Heart Rhythm. 2006;3(1):108–113. | **Verified** | Correctly cited. |
| 28 | Steptoe A, Kivimäki M. Stress and cardiovascular disease: an update on current knowledge. Annu Rev Public Health. 2013;34:337–354. | **Verified** | Correctly cited. |
| 29 | Lombardi F, Sandrone G, Mortara A, et al. Linear and nonlinear dynamics of HRV after acute MI with normal and reduced LVEF. Am J Cardiol. 1996;77(15):1283–1288. | **Verified** | Correctly cited. |
| 30 | Pencina MJ, D’Agostino RB Sr, Steyerberg EW. Extensions of net reclassification improvement calculations to measure usefulness of new biomarkers. Stat Med. 2011;30(1):11–21. | **Verified** | Correctly cited. |
| 31 | Huikuri HV, Mäkikallio TH, Peng CK, Goldberger AL, Hintze U, Møller M. Fractal correlation properties of R-R interval dynamics and mortality after acute MI. Circulation. 2000;101(1):47–53. | **Verified** | Correctly cited. |
| 32 | Stein PK, Domitrovich PP, Huikuri HV, Kleiger RE. Traditional and nonlinear HRV are each independently associated with mortality after MI. J Cardiovasc Electrophysiol. 2005;16(1):13–20. | **Verified** | Correctly cited. |
| 33 | Calderón-Juárez M, Cruz-Vega IB, González-Gómez GH, Lerma C. Nonlinear dynamics of heart rate variability after acutely induced myocardial ischemia by percutaneous transluminal coronary angioplasty. Entropy (Basel). 2023;25(3):469. | **CORRECTED** | Previous title/authors were wrong. Corrected per reviewer and verified (DOI 10.3390/e25030469). |
| 34 | Castelbuono S, Bellavia D, Sparacino L, et al. Autonomic impairment in advanced heart-failure patients revealed by nonlinear heart rate variability measured during exercise. Sci Rep. 2026;16(1):50905. | **CORRECTED** | Previous entry fabricated. Corrected per reviewer (DOI 10.1038/s41598-026-50905-4). |
| 35 | Kuzemczak M, Białek-Ławniczak P, Torzyńska K, et al. Comparison of baseline HRV in stable ischemic heart disease patients with and without stroke in long-term observation. J Stroke Cerebrovasc Dis. 2016;25(10):2526–2534. | **Verified** | Re-checked this round — authentic (title/volume/pages confirmed). |
| 36 | Kuzemczak M, Białek-Ławniczak P, Torzyńska K, et al. Correlation between HRV and severity of coronary atherosclerosis: looking for non-invasive indicators of the disease severity. World J Exp Med. 2026;16(1):111197. | **Verified** | Re-checked this round — authentic (DOI 10.5493/wjem.v16.i1.111197). |
| 37 | Lawler PR, Filion KB, Eisenberg MJ. Efficacy of exercise-based cardiac rehabilitation post-MI: a systematic review and meta-analysis. Am Heart J. 2011;162(4):571–584. | **Verified** | Correctly cited. |
| 38 | Premchand RK, Sharma K, Mittal S, et al. Autonomic regulation therapy via left or right cervical vagus nerve stimulation in chronic heart failure (ANTHEM-HF). J Card Fail. 2014;20(11):808–816. | **Verified** | Correctly cited. |
| 39 | Roest AM, Martens EJ, de Jonge P, Denollet J. Anxiety and risk of incident coronary heart disease: a meta-analysis. J Am Coll Cardiol. 2010;56(1):38–46. | **Verified** | Correctly cited. |
